# Results in a Series of Cases of Tonsillectomy at the Massachusetts General Hospital, Three to Four Years after Operation

**Published:** 1913-09

**Authors:** J. Payson Clark

**Affiliations:** Boston


### Results in a Series of Cases of Tonsillectomy at the Massachusetts General Hospital, Three to Four Years After Operation.

Postals were sent out in July, 1912, to patients who had been operated on in 1908, and 143 patients responded by presenting themselves to the clinic in person, where they were subjected to an examination and answered a set of questions with reference to the operation and after-effects. From these results the following summary is presented: The patients, with a few exceptions, were under fifteen years of age at the time of the operation.

Hemorrhage after tonsillectomy calling for special treatment was of rare occurrence. The condition for which the tonsils were removed was relieved in nearly every case, even in those in which there was some tonsillar tissue remaining. An improvement of the general health was to be expected after tonsillectomy done for such cause. Children who had had tonsillectomy certainly showed no increased tendency to illness and were probably less susceptible than before the operation. The present health of these children is excellent in the majority of cases. What is apparently tonsil tissue is found much more often than supposed after tonsillectomy. The soft palate was symmetrical and the faucial pillars and tonsil fossæ normal in the great majority of the cases. The accidental cutting off of the uvula in four cases caused no bad symptoms. Most of the cases of sore throat and tonsilitis were relieved by the operation. In many cases in which there appeared to be tonsil tissue remaining, the patients were in perfect health, and in others in which there were symptoms, those were undoubtedly due in many cases to causes other than the tonsil remnants. The ordinary voice or speech may be said to be practically unaffected by tonsillectomy. No investigation was made of the singing voice.

In most of the cases in which enlarged ce· vical glands could be felt, there was tonsil tissue present on the same side. In nearly half the cases in which there was tonsil tissue present, there were no enlarged glands. Carious teeth were apparently a direct cause of some cases of cervical adenitis.—Dr. J. Payson Clark, Boston.

